# Investigation into the animal species contents of popular wet pet foods

**DOI:** 10.1186/s13028-015-0097-z

**Published:** 2015-03-10

**Authors:** Isabella R Maine, Robert Atterbury, Kin-Chow Chang

**Affiliations:** School of Veterinary Medicine and Science, Sutton Bonington Campus, University of Nottingham, ᅟ, LE12 5RD United Kingdom

**Keywords:** Pet food, Species authentication, Labelling, food allergy, Ingredients, Animal proteins, DNA detection

## Abstract

**Background:**

The use of the generic term “meat and animal derivatives” in declared ingredient lists of pet foods in the European Union is virtually universal. In the wake of the 2013 “horse meat scandal” in the human food chain, we examined the presence and authenticity of animal sources (cow, chicken, pig and horse) of proteins in a range of popular wet pet foods in the United Kingdom.

**Findings:**

Seventeen leading dog and cat foods were sampled for the relative presence of DNA from each of the four animal species by quantitative real-time polymerase chain reaction. No horse DNA was detected. However, there was detection at substantial levels of unspecified animal species in most products tested. In 14 out of 17 samples, bovine, porcine and chicken DNA were found in various proportions and combinations but were not explicitly identified on the product labels. Of the 7 products with prominent headline descriptions containing the term “with beef”, only 2 were found to contain more bovine DNA (>50%) than pig and chicken DNA combined.

**Conclusions:**

There is a need for the pet food industry to show greater transparency to customers in the disclosure of the types of animal proteins (animal species and tissue types) in their products. Full disclosure of animal contents will (a) allow more informed choices to be made on purchases which are particularly important for pets with food allergies, (b) reduce the risk of product misinterpretation by shoppers, and (c) avoid potential religious concerns.

## Findings

In January 2013, the European meat industry was cast into the spotlight when the Food Safety Authority of Ireland (FSAI) announced that horse DNA was discovered in beef burgers sold in British and Irish supermarkets. Subsequently, large scale discrepancies between declared and actual meat contents came to light [1]. The pet food industry in the European Union operates under legislative guidelines and is largely represented by the European Pet Food Industry Federation (FEDIAF), which issues its own Code of Good Labelling Practice (FEDIAF, 2011) [2]. Similar checks on pet food authenticity and traceability appear not to have been reported to date.

To examine the correlation between composition of different animal proteins and the animal species that were disclosed on pet food labels, we determined the relative presence of DNA of cow, chicken, pig and horse in 17 leading dog and cat wet foods, which are readily available in UK supermarkets. This was achieved by the use of quantitative TaqMan real-time polymerase chain reaction based on genomic DNA derived from pet food contents that were presented in a variety of physical states. Genomic DNA was extracted using Qiagen QIAamp DNA Mini Kit in accordance with supplied instructions. The four animal species-specific primers and TaqMan probes, based on the 3’-untranslated region of the corresponding *myosin heavy chain slow* gene (Table 1), were validated with a panel of mammalian genomic DNA to be host species-specific (data not shown). A Cq standard curve with known amounts of genomic DNA was generated for each animal species. The relative amount of DNA detected for each host species was calculated as a percentage of total detected DNA. The presence of corresponding protein was implicit in the detection of animal species-specific DNA. The limitation of this study is that the presence of DNA from species other than cow, horse, pig and chicken would not have been recognised. However, it is reasonable to expect that animal proteins in pet foods are largely derived from cows, pigs and chickens.

**Table 1:** **Sequence design of primers and TaqMan probes for each animal species was based on the 3’-untranslated region of the corresponding** *myosin heavy chain slow* **gene**

**Host species**		
Cow	Forward	TGAGAGGGCACAGGACTTTCA
	Reverse	CAGTGGAGGCTGTGTCTGGAA
	Probe	CCGTAGCTTCATCCC
Horse	Forward	TGAGAGGCTGAGGACTTGCA
	Reverse	ATGGTGGTGGCCGTGTCT
	Probe	TGGCCAACTATCCTTC
Pig	Forward	CCCATGAGGCTCGGAACA
	Reverse	AGGAGGTGCAAGGAGCATGT
	Probe	CCTACTGCAACAATG
Chicken	Forward	GGGTGATAAGGAGGCCAGAAA
	Reverse	CACTACTTCCTGGTGCCAACAG
	Probe	AGCCCCATTCCTG

No horse DNA was detected in any of the 17 pet foods sampled. The relative abundance of bovine, porcine and chicken DNA in each pet food along with disclosed animal species information that accompanied each product is shown in Table 2. A major finding was the relative abundance of proteins from unspecified animal species in 14 of the 17 products. Amongst these 14 samples, bovine, porcine and chicken DNA were found in various proportions and combinations but were not explicitly named on the product labels. With two products (“Encore Chicken Breast with Brown Rice” and “Chappie Original”), the detection of chicken DNA only was consistent with their declared chicken contents. Two products (“Hill’s Prescription Diet R/D Feline Weight Loss” and “Cooperative Gourmet Terrine with Chicken and Game”) appear to contain no and 1% chicken DNA respectively, contrary to expectations.

**Table 2:** Protein information on product labels provided partial disclosure of animal species of origin

Each product shows headline label description and animal protein details provided by manufacturers. The relative amount of DNA detected for each host species tested for (listed in Table 1) was calculated as a percentage of total detected DNA. SD = standard deviation. *Fish DNA was not tested for in this study. The pie charts represent the proportion of the ingredients in relation to each other, and not the percentage of each ingredient in the whole product. Fish and turkey DNA were not tested in this study.

Another key observation concerned the headline description on pet food labels. Seven products with prominent descriptions containing the term “with beef” comprised between 14% and 56% bovine DNA. Only two of the seven were found to contain more bovine DNA (>50%) than pig and chicken DNA combined. With the remaining five samples, three contained more pig than bovine DNA. Another 6 headline labels that highlighted “chicken” or “with chicken” contained 1% to 100% chicken DNA of which two products contained more pig or bovine than chicken DNA.

The use of the category “meat and animal derivatives” in the ingredient list of pet foods is virtually universal. Given that the definition of “meat and animal derivatives” refers to “all products and derivatives of the processing of the carcase….of warm-blooded land animals” [2], the descriptive terms used in pet food labels, like “beef” and “chicken” do not necessarily mean bovine and chicken skeletal muscle but should be regarded as bovine and chicken protein derivatives respectively. There appears to be no legal requirement for a minimum level of skeletal muscle (meat) in pet foods. Furthermore, where a specific animal species is mentioned, such as “with beef”, it could constitute a minor component of total animal proteins in the product so long as it meets the minimum content of 4%.

Whilst the present practice in pet food labelling appears to be within current regulatory guidelines, our findings have highlighted two related weaknesses in product labelling that could adversely affect consumers (pets) and their owners. Firstly, in most products evaluated (14 out of 17), substantial protein contents of cow, pig and chicken were found but these animal species were not explicitly specified on product labels. Secondly, headline product information may not tally with buyers' expectations. Our findings highlighted what could be regarded as a considerable mismatch in the labelling standard of the pet food industry and what the purchaser might reasonably expect. For example, it would be reasonable for a purchaser to expect from a product prominently labelled as “beef stew” to have “beef” and not “chicken” as the major animal constituent. It may be a surprise to shoppers to discover that prominently described contents such as “beef” on a tin could, within the Guidelines, be a minor ingredient, have no bovine skeletal muscle (meat) and contain a majority of unidentified animal proteins.

It was recently reported that there was extensive presence of proteins from undeclared animal sources even in specially formulated commercial limited-antigen diets designed for canine adverse food reaction [3]. There is therefore a case for the pet food industry to show greater transparency to customers by fully specifying the different types of animal proteins (animal species and tissue types) in their products. UK human food laws already require meat products to be labelled with Quantitative Ingredient Declarations (QUID), which provide both the source and relative abundance of each meat ingredient [4]. Adopting such practices in pet food manufacturing will (a) allow more informed choices to be made on purchases which are particularly important for pets with allergies to certain animal proteins [5,6], (b) reduce the risk of product misinterpretation by shoppers, and (c) avoid potential religious concern as varying levels of undeclared pig DNA were found in five out of seven cat foods evaluated (Table 2).
